# Using simulation to explore medical students’ understanding of integrated care within geriatrics

**DOI:** 10.1186/s12909-019-1758-9

**Published:** 2019-08-28

**Authors:** Samantha Yang, Zarah Chaudhary, Maria Mylopoulos, Rida Hashmi, Yvonne Kwok, Sarah Colman, Thirumagal Yogaparan, Sanjeev Sockalingam

**Affiliations:** 1 MD Program, University of Toronto, 27 King’s College Cir, Toronto, ON M5S 3H7 Canada; 2 The Wilson Centre for Research in Education, Faculty of Medicine, University of Toronto, University Health Network, 200 Elizabeth Street, 1ES-565, Toronto, ON M5G 2C4 Canada; 3 Department of Psychiatry, University of Toronto, 250 College Street, 8th floor, Toronto, ON M5T 1R8 Canada; 4 Department of Family and Community Medicine, University of Toronto, 500 University Avenue, 5th floor, Toronto, ON M5G 1V7 Canada; 5 Centre for Addiction and Mental Health, 33 Russell Street, Suite 2065, Toronto, ON M5S 2S1 Canada; 6 Baycrest Centre for Geriatric Health Care, University of Toronto, 3560 Bathurst Street, Toronto, ON M6H 4A6 Canada

**Keywords:** Integrated care, Undergraduate medical education, Simulation

## Abstract

**Background:**

Given the increasing evidence and expansion of integrated care (IC) in healthcare, new IC curricula introduced early in undergraduate medical education (UME) are needed. Building on a pilot IC simulation called “Getting to Know Patients’ System of Care” (GPS-Care), we aimed to explore students’ understanding of patients’ complex physical and mental health needs, and to increase our understanding of how students learned in this simulation.

**Methods:**

177 of 259 first-year medical students participated in GPS-Care at the University of Toronto. Students role-played an elderly patient or caregiver within 5 simulated healthcare professional appointments. Students completed written reflections and 7 students participated in one-on-one interviews. A thematic analysis of the reflections and transcripts was conducted and descriptive data was generated for questionnaires.

**Results:**

Data saturation was reached at 43 reflections and 7 transcripts and the following themes emerged: a) students reflected on patients’ complex care experiences, b) students reflected on of the healthcare system needs care, c) students increased understanding of IC, and d) students desire to improve the care of IC patients within the healthcare system.

**Conclusions:**

In addition to confirming previous pilot study themes, the results from this study identified the role of productive struggle to provide students with a deeper understanding of patients’ IC care needs. Moreover, GPS-Care resulted in a transformative learning experience resulting in new insights into the importance of IC early in UME training.

**Electronic supplementary material:**

The online version of this article (10.1186/s12909-019-1758-9) contains supplementary material, which is available to authorized users.

## Background

Rapidly shifting global trends related to a rapidly aging population have resulted in concerns about workforce capacity in health care [1, 2]. Moreover, the high degree of psychiatric burden in older patient populations further adds to the chasm between the health care needs of an aging population and access to mental health services [3]. Data from the United States showed that 11.45 and 6.8% of older age patients had a past-year anxiety disorder and past-year mood disorder, respectively [3]. Given the potential impact of mental health on older adults’ overall health outcomes, there is a need for health care providers, including physicians, to be armed with the knowledge and skills to manage mental health conditions in geriatric patients [4].

Integrated care (IC) models have emerged as a potential care delivery solution to address the mental health needs of older adults presenting to primary care settings in the context of limited resources. There are several studies demonstrating increasing effectiveness of IC models, specifically the collaborative care model, for depression and anxiety [5–8] generally in adult patient populations. The potential for IC models to address the aging population’s health needs has resulted in an increased focus on IC training and capacity building across the learner continuum.

The American Psychiatric Association Council on Medical Education and Lifelong Learning has advocated for more educational experiences, including simulation, that focus on IC training and recommends that IC training begin in preclinical years to better prepare all future physicians for IC [9]. Dube and Verduin examined IC within undergraduate medical education (UME), and stated that despite increasing emphasis on IC “there is not a greater integration of behavioral health into other areas of the preclinical curriculum” [10]. Moreover, research also shows that adoption of the IC model is still slow, especially in academic medical centers [11]. Therefore, there remains a need for IC curriculum offered early in medical school to foster strong skills in this area for future physicians.

To respond to the limited UME curricula focused to date on IC training, in 2016, a pilot curriculum consisting of an interprofessional simulation experience with 20 preclinical medical students called ‘Getting to Know Patients systems of care (GPS-care experience)’ was implemented at the University of Toronto as part of preclinical curriculum renewal [12]. This novel simulation approach to IC education provided students with a patient’s perspective on the experience of co-ordination and navigation of their health care when suffering from co-morbid health, namely diabetes, and psychiatric conditions. The reflections and evaluations of this pilot GPS-Care experience showed that students felt increased connection and empathy towards patients, improved understanding of patient related determinants of health and a greater understanding of patient navigation of the current health care system as well as increased motivation to tackle challenges faced by patients [12].

While the GPS-Care pilot showed an impact on student learning, the evaluation did not clarify why the simulation was effective. The concept of productive struggle offers a potential mechanism by which GPS-care increases students’ understanding of navigation of care challenges experienced by patients with both physical and mental health conditions [13–15]. Productive struggle consists of experiences where students first engage in problem solving before receiving instruction on underlying concepts, which is then followed by instruction to consolidate learning [13–15]. For example, in GPS-Care students participate in multiple simulated experiences where they experience the challenges of how care is delivered for patients with co-occurring physical and mental health needs in different interprofessional contexts. After this experience, students receive instruction through a facilitated debrief and reflection post-event to further guide learning [16].

Although the conceptual framework of productive struggle might support a deeper learning of IC, data has not fully examined this concept in GPS-Care. Therefore, we used an expanded GPS-Care education initiative (*n* = 259) at the University of Toronto to examine this potential mechanism for learning in GPS-Care and to build on preliminary themes from the pilot data.

In this paper, we aim to further explore students’ understanding of the care needs and challenges of complex patients by means of an expanded simulation experience during their preclinical UME year. Building on our initial findings in the GPS-care experience pilot, we aim to further explore if the productive struggle resonated with students’ experiences and learning.

## Methods

### Design and participants

The GPS-Care experience consisted of medical students role-playing a geriatric patient or one of their two caregivers navigating through a simulated healthcare system. Five patient and caregiver scenario stems were developed by a team of content experts and clinician reviewers. Each narrative consisted of the care experience of a geriatric patient having comorbid mental and physical conditions and the roles and experiences of their two caregivers (Additional file 1 for sample summary of scenario).

**Fig. 1:**
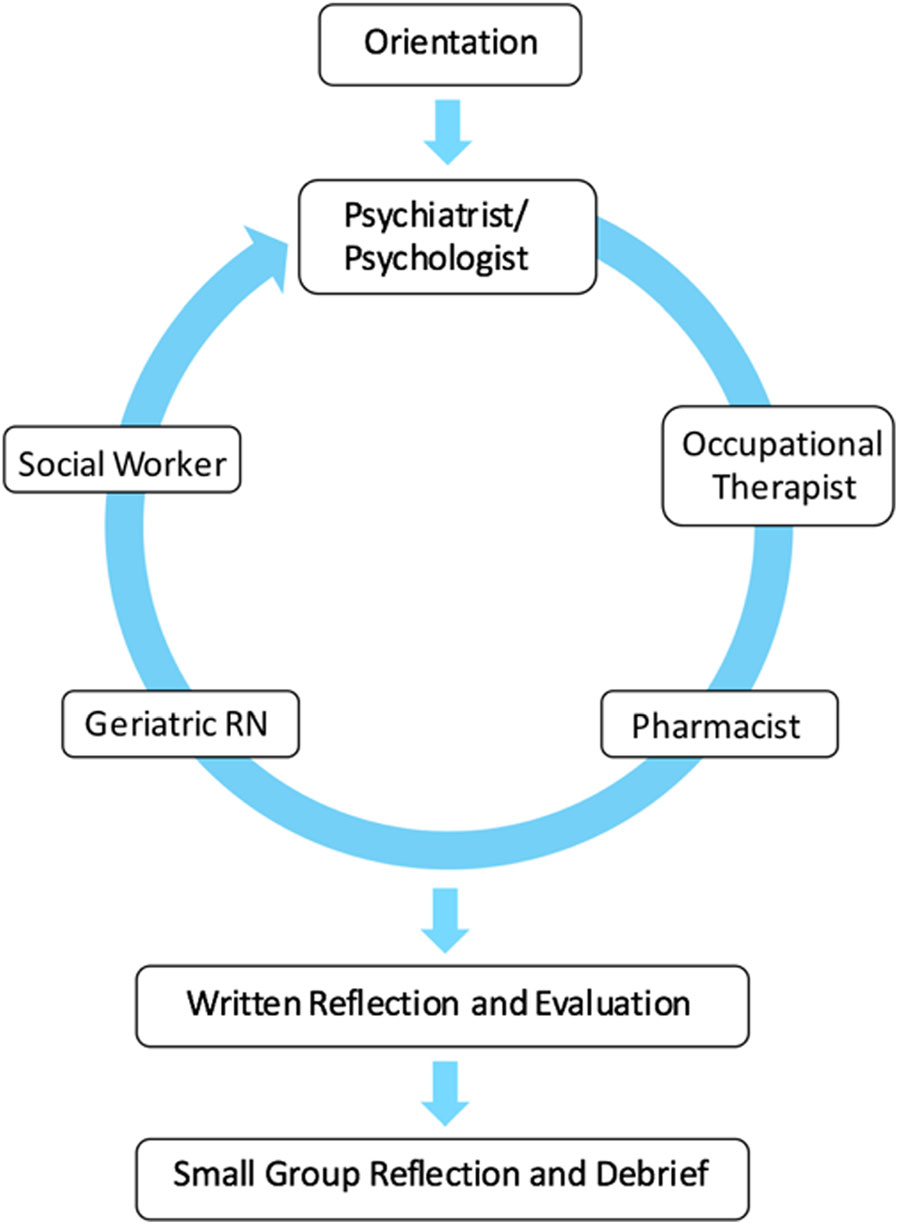
Illustrating the patient and caregiver role-play experience. After the students were oriented to the experience, they were assigned to role-play either a patient or caregiver and read their given scenarios. They subsequently attended 5 simulated appointments with healthcare professionals in their assigned roles. After the experience, students completed a written reflection and evaluation and then attended a small group reflection and debrief

In groups of three, students followed the patient or caregiver narratives by attending five simulated appointments with the following healthcare professionals (HCPs): geriatric nurse, psychiatrist or psychologist, occupational therapist, social worker, and community pharmacist. The patient’s case also included a description of follow up provided by their primary care physician (PCP) between each appointment (Fig. 1). Stems were provided at each station detailing the evolution of the patient’s physical and mental health, the caregiver’s concerns, and follow-up from the PCP.

From a class of 259 first-year medical students at the University of Toronto, 177 students were randomly selected to participate in GPS-Care as part of a 2 year implementation of this mandatory training program. Participation in GPS-Care consisted of each student receiving a package outlining their patient or caregiver character and participating in a 15 min orientation session before proceeding to their respective stations to start the experience. The entire simulation including multiple appointments and care experiences lasted 1.5 h and the experience concluded with a 45-min formal group debrief session led by a facilitator.

All students completed a written reflection that explored their perceptions on the roles of various HCPs, patient belief systems, and navigating the healthcare system (see Additional file 2). Questions sought to capture what knowledge was exchanged between HCPs and patients or caregivers, critical learning moments, and changes in students’ attitudes and perceptions regarding the care of patients with co-occurring physical and mental health conditions. Lastly, individual interviews were conducted with students who consented to be contacted after their GPS-Care participation within 3 months after the experience. Ethics approval was obtained from the Health Sciences Research Ethics Board at the University of Toronto and participating students provided written informed consent.

### Data analysis

A total of 43 written reflections were used for analysis before reaching data saturation. Seven participants consented to participate in an additional one-on-one interview about their learning outcomes and received a gift card for their time. A qualitative thematic analysis of the students’ written reflections and post-GPS-care interview transcripts was conducted to explore students’ experiences navigating through the various appointments in the geriatric role-play. Seventy-four pages of typed student reflections and interview transcripts were anonymized and responses were considered in aggregate. SY (a second-year medical student researcher) performed open coding and analyzed the data inductively in June–August 2017 in consultation with an expert qualitative education scientist (MM) and ZC (an education research associate with a background in learning and memory research who had conducted pilot analyses) over 3 meetings. During these meetings, these codes and categories were considered with respect to data from the pilot study [12] and iteratively consolidated a thematic framework that incorporated the novel themes stemming from the new geriatric context. The framework was developed by systematically integrating categories using the constant comparative method within and between datasets [17]. SY then performed another round of coding the dataset, this time using the consolidated framework deductively, for verification of coverage. An audit trail capturing the successive iterations of analyses was maintained. Survey data was analyzed using a descriptive analysis.

## Results

A total of 177 students participated in this GPS-Care experience and students reported perspectives on patient care, navigating the healthcare system, the utility of IC, and sought to implement their learnings in future practice. The thematic analysis of student reflections and individual interviews yielded four major themes described below.

### Theme 1: students reflected on the experiences of complex geriatric patients

Students reported that singular issues are challenging to identify in patients with multiple physical and mental conditions and therefore, should be treated concurrently because “*one does not outweigh the other”* (Table 1, Subtheme 1.1). They recognized that patients with cognitive impairment may lack self-awareness, have difficulty maintaining their health, and consequently rely on their caregivers for support. Students reported the importance of focusing on patient needs and values, and tailoring care to each patient (Table 1, Subtheme 1.2). They went beyond understanding the patient perspective by identifying the desire for caregivers to express their beliefs and devised means to keep their patient’s values in the forefront while managing the views of the caregivers. When students role-played as patients, they identified feeling disheartened when their perspectives were ignored and that it made them feel like they were losing their independence (Table 1, Subtheme 1.3). Overall, students reflected on the challenges of treating patients with several physical and mental illnesses, the importance of the patient perspective, and how dismissing the concerns of patients can be frustrating for them (see Table 1 for subthemes and Additional file 3 for relevant quotes).

**Table 1:** Subthemes within thematic framework of results

Theme 1. Students reflected on the experiences of complex geriatric patients	
1.1 Students reported an increased understanding of the challenges of co-occurring physical and mental health needs and how psychosocial issues can contribute to health, as well as one’s experience navigating the system	
1.2 Students reported increased appreciation of individual patients’ values, culture and belief systems, autonomy, as well as patients’ understanding of their own physical and mental health	
1.3 Students expressed frustrations when their views and needs as patients were dismissed	
Theme 2. Students reflected on the system of care	
2.1 Students expressed an understanding of the proposed value of an interprofessional support system	
2.2 Students reported that the interprofessional system was overwhelming when not effectively coordinated	
2.3 Students identified psychosocial factors, belief systems, and logistics of care as important barriers to accessing healthcare	
2.4 Students reflected on the goals of the healthcare services provided by the HCPs and the healthcare system as a whole	
Theme 3. Students increased their understanding of integrated care after struggling with care navigation	
3.1 Students identified the act of managing extensive multi-source information and a cognitive overload of information as primary challenges to understanding patient conditions and the implementation of care plans	
3.2 Students reported increased understanding of the challenges of navigating the healthcare system for patients with co-occurring physical and mental health needs with particular emphasis on the importance of catering to individualized psychosocial needs	
Theme 4. Students aspired to facilitate care integration	
4.1 A central aspiration among students was to adapt to and integrate the different physical, mental, and psychosocial needs of their patients to provide holistic patient care	
4.2 Students discussed the need to spend more time building a therapeutic alliance with their patients where they respect patient autonomy, perspectives, and decision-making	
4.3 Students identified learning more about community and government resources to better address psychosocial needs	
4.4 Students described the importance of adopting communication skills that will allow them to achieve their proposed framework of care	

### Theme 2: students reflected on the system of care

Students gained appreciation for interdisciplinary teams after interacting with HCPs and learned about the roles different HCPs occupy (Table 1, Subtheme 2.1). Consequently, students aspired to be more knowledgeable about community resources in order to utilize them in the future. Students recognized there was a drastic difference in quality of care with effective interprofessional communication compared to weak communication. They found that poor coordination was very frustrating and impacted the timing of appointments (Table 1, Subtheme 2.2). Students realized accessing care was affected by the support of caregivers, patient motivation, and ability to travel to visit HCPs (Table 1, Subtheme 2.3). Without access to care, referring patients to healthcare resources becomes irrelevant. Finally, students recognized that there was some overlap across HCP roles and although learning about overlapping resources felt redundant to some, others appreciated how each profession took a different approach to solving the same problem (Table 1, Subtheme 2.4). In summary, students reflected on the coordination of care in interprofessional teams, the roles of various HCPs, and the barriers patients experience when navigating the healthcare system (see Additional file 3 for relevant quotes).

### Theme 3: students increased their understanding of integrated care after struggling with care navigation

Students recognized that managing multiple co-morbidities of an older patient can be overwhelming due to an overload of information and terminology (Table 1, Subtheme 3.1). During the simulation, students received many recommendations from the HCPs, and struggled to recall information from the case after the simulation. As a result, they aspired to disseminate information in a more accessible way for their future patients. Students identified addressing the psychosocial needs of geriatric patients as an important part of providing holistic care, but the development of a treatment plan that balances the preferences of the patient and caregivers can be challenging (Table 1, Subtheme 3.2). Caregivers were viewed as vital components to patient care and students sought to support them in addition to the patient. Overall, students identified how IC may help manage issues such as overloading patients with information and failing to manage psychosocial issues that were identified by students (see Additional file 3 for relevant quotes).

### Theme 4: students aspired to facilitate care integration

Following the facilitated reflection post-GPS Care, students aspired to facilitate integration and developed their own approaches to meeting complex patients’ needs. Students realized that caregivers oversee the management of patients’ health and recognized the importance of the patient-caregiver relationship in providing holistic care (Table 1, Subtheme 4.1). Caregivers may be limited by time or finances and it is important to select a therapy that meets their needs in addition to the patient’s needs. Although managing appointments with patients and multiple caregivers can be difficult for physicians, after the simulation, students realized that patients really valued physicians who spoke directly to them and were willing to develop a therapeutic physician-patient relationship (Table 1, Subtheme 4.2). Through interacting with various HCPs, students learned about resources they can refer their patients to and consequently, sought to expand their knowledge of community services and coordinate their future referrals by time and location for patient ease (Table 1, Subtheme 4.3). Through the simulation, students experienced strong and substandard examples of interprofessional communication and recognized the negative effects of poor communication on patient care. By way of these negative experiences, students realized that communication is important between various HCPs and between the physician and the patient, especially when describing goals of treatment with patients (Table 1, Subtheme 4.4). Critically, after experiencing GPS-Care and learning about the complexities of geriatric patients, students aspired to integrate the care of these patients, validate their beliefs and autonomy, recommend community resources when appropriate, and be strong communicators to better care for this population (see Additional file 3 for relevant quotes).

## Discussion

The current study with the new focus of the GPS-Care simulation experience in a geriatric setting replicated many of the findings from our smaller 20-person pilot study of GPS-Care [12]. That is, students reflected on navigating the healthcare system from the perspective of a geriatric patient with co-occurring physical and mental health illnesses and on the system of care (Themes 1–2). Additionally, the current study sought not only to understand students’ experiences and learning but to explore *why*, or the mechanism by which students came to effectively understand IC concepts such as team-based care, care navigation and an appreciation of the impact of co-occurring physical and mental health within a geriatric patient population. We found that as students ‘struggled’ with care navigation they came to have a greater understanding of integrated care (Theme 3). Our data suggest that the process of engaging students in the GPS-Care as patients striving to have their needs met was a process instrumental to increasing students’ awareness of some IC concepts prior to receiving guided instruction on these underlying concepts during debrief. For example, students identified that a successful clinician was able to balance the physical and mental health comorbidities of the patient while addressing the patients’ needs and concerns [18]. An unexpected finding was students’ aspirations to improve patients’ access to healthcare and facilitate integration in several ways in the future (Theme 4), such as by communicating more effectively with other HCPs. Therefore, GPS-Care provides a simulation experience to potentially address previously identified gaps in IC UME training [9].

Taken together, data across the four themes are in line with the literature on the productive value of “struggling” with a challenging experience on students’ learning of target underlying concepts [13, 15]. That is, students ultimately understood the critical components of integrated care by first struggling with the challenging task of navigating a fragmented system and reflecting on the problems faced by patients and their caregivers; and then having a facilitator help them build on their representations of the system of care based on their experience. Therefore, this study underscores the importance to providing early experiences that allow students to be challenged by exposure to crucial facets of IC in medical school before receiving instruction on these concepts to consolidate learning.

Beyond an increased understanding of integrated care concepts, an unexpected finding was a significant shift in students’ understanding of the system of care and care coordination (Theme 4), such that it instilled in them a desire for creating change. Specifically, experiencing suboptimal navigation and sometimes inadequate care, critically reassessing their presuppositions on care navigation for complex patients empowered students and instilled in them a desire to behave differently i.e. spending more time with the patient and articulating their own approaches to meet the needs of complex patients. This may suggest that components of the GPS-Care experience are in line with critical pedagogical practices that can support or facilitate accretions of transformative learning. This kind of learning involves a disorienting experience that provokes a fundamental shift in a “frame of reference” or schema through the process of critically reflecting on epistemic assumptions from prior learning. These meaning schemes and beliefs then guide the learner to action such as acquiring new skills, attitudes or behaviours [19–22]. While it is unclear which preconditions specifically triggered this shift, objective reframing has been proposed to support this type of learning. Guiding learners to become critical of their own frames of reference on the system of care by having them be engaged in a role-playing activity that reframed the problem using a different perspective [20] may have created a cognitive disequilibrium that propelled students to construct new schemas and alternative perspectives [23]. Future work could further explore these processes and how they intersect with pedagogical approaches like ‘guided discovery’ to further elucidate their role in producing significant deep learning.

While similar simulation studies have been conducted with pharmacists, nursing students and general practice receptionists with patient scenarios and standardized patients [24–26], the complexity of the geriatric patient, inclusion of both patient and caregiver roles, and rotations between appointments with various HCPs contributed to a richer understanding of IC. In particular, the caregiver roles in this simulation experience provided additional insights into IC that have not been previously observed or described in the IC education literature. In struggling to have the needs of the patient understood during encounters with providers, students gained an appreciation for the unique role that caregivers played in a poorly integrated system. This enabled students to recognize and acknowledge the significance of including caregivers in decision-making and to empathize with them. Caregivers have been previously recognized for playing a central role in integrating patient care across multiple HCPs, which was supported by the data from GPS-Care [27].

Our study findings should be considered in the context of the following limitations. First, the study was conducted in one institution so GPS-Care could be tested elsewhere for generalizability. Another limitation is that the research participants volunteered their time, representing a subset of medical students, and possibly introducing selection bias. In addition, this is a cross-sectional study and future studies should utilize longitudinal designs to see if there are long-term changes in learning outcomes. In the future, we will ensure ongoing exposure of integrated care for the students who participated in the simulation throughout their undergraduate curricula.

## Conclusion

Increasing evidence supporting IC models have led to an increased focus towards using them to address the physical and mental health needs of geriatric patients. The GPS-Care experience has shown to be an effective way of introducing IC early in medical training by way of engaging students in a challenging experience followed by instruction on core concepts of IC. The experiences of productive struggle in GPS-Care potentially fostered a transformative learning experience, specifically their desire to change their future IC practice.

## Additional files

Additional file 1: Sample summary of scenario. (DOCX 13 kb)

Additional file 2: Reflection Questions. (DOCX 15 kb)

Additional file 3: Relevant Quotes. (DOCX 17 kb)

## Data Availability

The data analysed during the current study are not publicly available but are available from the corresponding author on reasonable request.

## Abbreviations

GPS-Care: “Getting to Know Patients’ System of Care”
HCPs: Healthcare professionals
IC: Integrated care
PCP: Primary care physician
UME: Undergraduate medical education
