# Supplementary material for: Using simulation to explore medical students’ understanding of integrated care within geriatrics
Source: BMC Med Educ. 2019 Aug 28;19:322. doi: 10.1186/s12909-019-1758-9 (PMC6712598; doi:10.1186/s12909-019-1758-9)
Supplement: Supplementary file 1 — Sample summary of scenario. (DOCX 13 kb) [file 12909_2019_1758_MOESM1_ESM.docx]

**Supplementary Material S1.** Sample summary of scenario.

An example of a case is a 65-year old woman who suffered from stroke that caused her to move in with her daughter, husband, and their 3 children. The patient experiences memory issues, multiple minor falls, feels isolated during the day, and struggles with her loss of independence. The daughter struggles with the stress of taking care of her mother and her kids, occasionally snapping at her husband. The husband supports his wife and mother-in-law to the best of his ability and often mediates conflicts.
