# Supplementary material for: Using simulation to explore medical students’ understanding of integrated care within geriatrics
Source: BMC Med Educ. 2019 Aug 28;19:322. doi: 10.1186/s12909-019-1758-9 (PMC6712598; doi:10.1186/s12909-019-1758-9)
Supplement: Supplementary file 2 — Reflection Questions. (DOCX 15 kb) [file 12909_2019_1758_MOESM2_ESM.docx]

**Supplementary Material S2.** Reflection Questions.

1. What roles did the different health care professionals play in your care and what information did they offer?
2. Following this role-playing, what are your perceptions on the value of a patient’s belief systems, their preferences and their culture context in meeting their needs? In medical decision-making? Did these perceptions previously differ?
3. What did you learn about medical psychiatry patients’/caregivers’ experience in navigating their care in the community?
   1. What was helpful?
   2. What was challenging?
4. What did you learn about the care experience for patients with co-occurring physical and mental health (psychosocial) conditions?
   1. What about timeliness of care?
   2. What about continuity of care & care coordination?
5. Did your perceptions about the management of both physical and mental health issues change as a result of this role-play experience? If so, how? If not, how come?
6. What was the most valuable thing that you learned today? What was an important insight that you had during this experience? Can you identify a key moment that affected your care (ex. A moment where either something unexpected happened or you felt uncomfortable etc.)?
7. Was there ever a moment when you felt you didn’t get trusted care or you struggled with your interaction with your healthcare provider (i.e. where your needs were not being met)?
8. How has this experience influenced what kind of physician you would hope to become?
