# Supplementary material for: Using simulation to explore medical students’ understanding of integrated care within geriatrics
Source: BMC Med Educ. 2019 Aug 28;19:322. doi: 10.1186/s12909-019-1758-9 (PMC6712598; doi:10.1186/s12909-019-1758-9)
Supplement: Supplementary file 3 — Relevant Quotes. (DOCX 17 kb) [file 12909_2019_1758_MOESM3_ESM.docx]

**Supplementary Material S3.** Relevant Quotes.

| **Table 3. Subthemes of Theme 1 with Relevant Quotes** | |
| --- | --- |
| **Subthemes** | **Quotes** |
| 1.1 Students reported an increased understanding of the challenges of co-occurring physical and mental health needs and how psychosocial issues can contribute to health, as well as one’s experience navigating the system | “*It is often difficult to distinguish between physical and/or mental health conditions. In our case, confusion could be due to a physical problem (i.e. past stroke, head injury) or a mental problem (i.e. depression). It is important to always ask about the psychosocial aspects of a patients care and there is overlap between physical & mental health aspects of care.”* |
| 1.2 Students reported increased appreciation of individual patients’ values, culture and belief systems, autonomy, as well as patients’ understanding of their own physical and mental health | “*Perhaps the ideal would be some time alone with the patient to kind of talk about those things and then also having you know, the family there, the caregivers there, so they can also provide their input.”* |
| 1.3 Students expressed frustrations when their views and needs as patients were dismissed | “*I would say the only frustrating thing was that sometimes it seemed like, it was more like, they were telling us what they did and what they wanted to do and less of what they thought we wanted to accomplish, so maybe there was a bit of dissonance there.”* |

| **Table 4. Subthemes of Theme 2 with Relevant Quotes** | |
| --- | --- |
| **Subthemes** | **Quotes** |
| 2.1 Students expressed an understanding of the proposed value of an interprofessional support system | “*If I were a family doctor I would try to have all these resources available and perhaps have, have like a nurse in the clinic who could direct people to these resources … ideally, I would like to do it myself, but I just know that it would be difficult to find enough time to go through the resources for each person.”* |
| 2.2 Students reported that the interprofessional system was overwhelming when not effectively coordinated | “*I learned how slow the process can be, and how inconsistent the communication can be between professionals. … Having to constantly repeat info, being confused about next steps, unsure who should take care of follow-up ... I hope to be more empathetic when patients are angry/frustrated about their care, and that I will make arranging and checking follow-up a priority.”* |
| 2.3 Students identified psychosocial factors, belief systems, and logistics of care as important barriers to accessing healthcare | “*I think they were advocates for me getting the care I … so because of that I was able to get access to more services. And also as a patient it’s quite tiring to do it all on your own so I think it’s really important to have a support system in place.”* |
| 2.4 Students reflected on the goals of the healthcare services provided by the HCPs and the healthcare system as a whole | “*There was a lot of overlap that I found frustrating, as the social worker, OT, and geriatric nurse all suggested a day program, PSW, and meals on wheels. … It was interesting to see how different professions focused on different aspects of the story and how to help, like the OT providing shower rails and the pharmacist exploring hypoglycemia – both to address the same fall.”* |

| **Table 5. Subthemes of Theme 3 with Relevant Quotes** | |
| --- | --- |
| **Subthemes** | **Quotes** |
| 3.1 Students identified the act of managing extensive multi-source information and a cognitive overload of information as primary challenges to understanding patient conditions and the implementation of care plans | “*Remembering all the discussion that happened. … As an old woman I would have been very overwhelmed and found it hard to juggle all the suggestions provided.”* |
| 3.2 Students reported increased understanding of the challenges of navigating the healthcare system for patients with co-occurring physical and mental health needs with particular emphasis on the importance of catering to individualized psychosocial needs | “*And also, taking, really taking the time to see how the caregivers are doing because in my interviews it felt like the focus was on primarily on me, but I think in terms of the most holistic care, I think it would have been more helpful if there was lot of, if there was a bit more focus on how my caretakers were doing and what was best for them.”* |

| **Table 6. Subthemes of Theme 4 with Relevant Quotes** | |
| --- | --- |
| **Subthemes** | **Quotes** |
| 4.1 A central aspiration among students was to adapt to and integrate the different physical, mental, and psychosocial needs of their patients to provide holistic patient care | “*I don’t think it has changed my career path, but impacted the different considerations I will have of how treatments will impact families and those who the patient relies on. There is more concern to be had for relationships and support, not just biological problems.”* |
| 4.2 Students discussed the need to spend more time building a therapeutic alliance with their patients where they respect patient autonomy, perspectives, and decision-making | “*I hope to become a doctor that does not allow myself to feel rushed or pressured in a way that takes away from the care of my patients & their family.”* |
| 4.3 Students identified learning more about community and government resources to better address psychosocial needs | “*I think that I, I would find some way to help the patient keep the appointments straight and keep them in a very close location. For example, I know social workers often help with acting as a liaison between the patient and different healthcare providers. So if, if that was something that was applicable, I would find a social worker for the patient and when booking appointments or referring a patient to different services.”* |
| 4.4 Students described the importance of adopting communication skills that will allow them to achieve their proposed framework of care | “*I want to be an effective collaborator and communicator not only with my peers in the health care system but also my patients. I must listen to their [cents] and needs when creating a patient-centered treatment plan.”* |
